# Assessing Public Interest Based on Wikipedia’s Most Visited Medical Articles During the SARS-CoV-2 Outbreak: Search Trends Analysis

**DOI:** 10.2196/26331

**Published:** 2021-04-12

**Authors:** Jędrzej Chrzanowski, Julia Sołek, Wojciech Fendler, Dariusz Jemielniak

**Affiliations:** 1 Department of Biostatistics and Translational Medicine Medical University of Łódź Łódź Poland; 2 Department of Pathology Medical University of Łódź Łódź Poland; 3 Management in Networked and Digital Societies Kozminski University Warszawa Poland

**Keywords:** COVID-19, pandemic, media, Wikipedia, internet, online health information, information seeking, interest, retrospective, surveillance, infodemiology, infoveillance

## Abstract

**Background:**

In the current era of widespread access to the internet, we can monitor public interest in a topic via information-targeted web browsing. We sought to provide direct proof of the global population’s altered use of Wikipedia medical knowledge resulting from the new COVID-19 pandemic and related global restrictions.

**Objective:**

We aimed to identify temporal search trends and quantify changes in access to Wikipedia Medicine Project articles that were related to the COVID-19 pandemic.

**Methods:**

We performed a retrospective analysis of medical articles across nine language versions of Wikipedia and country-specific statistics for registered COVID-19 deaths. The observed patterns were compared to a forecast model of Wikipedia use, which was trained on data from 2015 to 2019. The model comprehensively analyzed specific articles and similarities between access count data from before (ie, several years prior) and during the COVID-19 pandemic. Wikipedia articles that were linked to those directly associated with the pandemic were evaluated in terms of degrees of separation and analyzed to identify similarities in access counts. We assessed the correlation between article access counts and the number of diagnosed COVID-19 cases and deaths to identify factors that drove interest in these articles and shifts in public interest during the subsequent phases of the pandemic.

**Results:**

We observed a significant (*P<.*001) increase in the number of entries on Wikipedia medical articles during the pandemic period. The increased interest in COVID-19–related articles temporally correlated with the number of global COVID-19 deaths and consistently correlated with the number of region-specific COVID-19 deaths. Articles with low degrees of separation were significantly similar (*P*<.001) in terms of access patterns that were indicative of information-seeking patterns.

**Conclusions:**

The analysis of Wikipedia medical article popularity could be a viable method for epidemiologic surveillance, as it provides important information about the reasons behind public attention and factors that sustain public interest in the long term. Moreover, Wikipedia users can potentially be directed to credible and valuable information sources that are linked with the most prominent articles.

## Introduction

After the COVID-19 pandemic outbreak began, a new concern for public health emerged—predicting and preventing the spread of the disease. The increased media coverage on the COVID-19 pandemic focused the public’s attention and likely affected the most popular internet search terms, thus altering people’s behavior worldwide [1,2]. The public consumption of COVID-19 information in digital media is directly associated with preventive behaviors, including regularly washing hands with soap and water, staying away from crowded places, and wearing face masks in public [3]. Bragazzi et al [4] have suggested that internet search trend data can be used to build predictive models of disease spread and help with containing the pandemic. Bento et al [5] has shown that the internet search term “coronavirus” increased in popularity immediately after the day of the first COVID-19 case announcement. However, the term’s popularity returned to the baseline level in less than 1-2 weeks. After this period, other terms that pertained to community-level policies (ie, quarantine, school closures, and COVID-19 tests) or personal health strategies (ie, masks, grocery delivery, and over-the-counter medications) emerged as the most-searched terms [5]. Other studies have shown that public interest in specific search terms is more associated with reported deaths and media coverage than with real epidemiologic situations [6]. Moreover, it has been observed that people quickly experience information overload, which results in information avoidance [7]. Therefore, it seems logical to identify the most credible and reliable sources and use them to inform the public during the “information-hungry” period of any subsequent pandemics that may occur.

Wikipedia is considered a key web-based source of health information, and people are more willing to seek information that is published in Wikipedia than information from any other health websites [8]. Wikipedia’s quality has been a highly debated topic, and many researchers are highly skeptical of the platform [9]. Nevertheless, in 2005, a comparative study that was published in *Nature* reported that Wikipedia was competing head-to-head with Britannica [10]. Since then, many different studies on Wikipedia's accuracy have shown that it is quite on par with published professional sources, as it provides reliable information [11,12]. Studies have reported that the quality of medical information that is available in Wikipedia is consistently high [13,14]. It has also been reported that Wikipedia is more reliable than several published sources, despite its low readability [15]. Medical articles in Wikipedia are based on reliable sources and mainly skew toward the most prominent academic journals [16]. More and more scholars have embraced the use of Wikipedia in the classroom [17,18]. The wide acceptance of Wikipedia is also related to institutional recognition. The American Psychological Association and the Association for Psychological Science have encouraged their members to edit Wikipedia articles [19,20].

With regard to medical information, Wikipedia's popularity exceeds that of the National Health Service, WebMD, Mayo Clinic, and World Health Organization (WHO) websites combined. This is mostly due to the highly accurate information that is provided by the editors of Wikiproject Medicine [21]. Wikiprojects are edited by self-organized groups of volunteers who are involved in curating information on a specific topic [13]. Since their reliability in providing medical information is so high, the WHO has partnered with the Wikimedia Foundation to expand access to trusted COVID-19–related information on Wikipedia [22]. Several studies have indicated that medical students who use Wikipedia to prepare for their exams receive better grades than students who only rely on textbooks [23]. Wikipedia has become a vital tool for global public health promotion [24].

The quality and popularity of Wikipedia's medical content make analyzing the most popular medical articles extremely interesting from a medical professional’s point of view. Additionally, changes in viewership and peaks in interest are of high relevance to public health specialists. Given that Wikipedia has over 300 language variations that differ significantly in terms of rules, culture, and the presentation of knowledge [25], it is also interesting to compare changes in the popularity of Wikipedia medical articles across languages. Hence, to better understand public interest in high-quality health information during the COVID-19 pandemic, we conducted an analysis of daily visits to Wikipedia articles that were published from 2015 to 2020 and sought to identify possible factors for attracting public attention.

## Methods

### Detecting and Quantifying the Surge in COVID-19–Related Searches

We collected a list of 37,880 articles that were curated by the English Wikipedia Medicine Project. We derived the daily access counts (ie, from July 1, 2015, to September 13, 2020) of these articles by using ToolForge (ie, a pageview analytics tool). Daily access was defined as each visit to a given article on a given date. These data did not contain user-specific information.

We limited our selection to the 100 most accessed English Wikipedia Medicine Project articles that were published from July 1, 2015, to September 12, 2020. No other filters were applied to included Wikipedia articles. By using an interwiki mechanism (ie, cross-references for different language versions of Wikipedia), we identified articles that reported on the same subject in nine other language versions of Wikipedia (ie, French, German, Swedish, Dutch, Russian, Italian, Spanish, Polish, and Vietnamese). The articles were matched, and their daily access counts from July 1, 2015, to September 13, 2020 were obtained. These data were stored in an Excel sheet that used English Wikipedia article names. Extracted daily access data are provided in Multimedia Appendix 1.

To provide additional context with regard to the ongoing COVID-19 pandemic, we also obtained pandemic-related data. We decided not to use reports on the total number of daily confirmed SARS-CoV-2 infection cases due to the possible influencing effect of introducing different public testing schemes. Other factors, such as the total number of daily deaths, are less likely to be altered by regional politics and health care discrepancies. Thus, we decided to measure the effect of the COVID-19 pandemic by analyzing the total number of global deaths and region-specific deaths, which were defined as the cumulative number of deaths resulting from SARS-CoV-2 infection for all reported countries and language-specific countries, respectively. These data were obtained from standardized public information in the Our World in Data University of Oxford initiative website [26].

The preprocessing of Wikipedia article access data was performed in Python 3.8, Excel version 2011 (Microsoft Corporation). Due to the influences of day-to-day differences in Wikipedia access and absolute differences in access to the different language versions of Wikipedia, we standardized access data by calculating daily article access as percentages of language-specific access to Wikipedia on a given day.

To identify deviations in the stability of article visits, we chose articles that exhibited the least altered access patterns throughout the investigated period. Articles were selected based on their relative stability (ie, the SD of percent article access in a 30-day moving window divided by the 30-day moving average [ie, mean] of percent article access), which was calculated for the full duration of investigated period. Relative stability was assessed to identify articles that exhibited relatively unchanged access patterns throughout the studied period. Reference articles were defined as the 20 most stable articles across all languages. These articles provided the highest mean daily access percentages for all evaluated periods. As such, a reference article could be used for direct comparisons with other highly accessed articles. They also provided metrics that were the least affected by changes in Wikipedia use.

### Statistical Analysis

A statistical analysis was conducted to identify access patterns in Wikipedia and Wikipedia Medicine Project articles that were published in 2020 and to determine these patterns’ association with the COVID-19 pandemic. First, we investigated the association between Wikipedia access before and during the COVID-19 pandemic. Second, we determined whether there were specific articles of interest before and during the COVID-19 pandemic (ie, excluding the years that were associated with other epidemics that were covered in media). We also investigated whether the total number of global and regional deaths resulting from SARS-CoV-2 were associated with increased access to articles of interest during the COVID-19 pandemic.

We also investigated whether there was a difference between navigation to Wikipedia articles of interest before and during the COVID-19 pandemic. To this end, we determined the minimum number of links required to navigate between two articles within Wikipedia (ie, the degree of separation [DOS]).

The statistical analysis was conducted in Python 3.8 and Statistica 13.3 (Statsoft, TIBCO Software Inc, Dell Inc). The DOS was assessed by using the PHP (hypertext preprocessor) language and jQuery library, which were implemented in the Degrees of Wikipedia tool [27]. To facilitate the straightforward representation of DOS results, we provided the DOSs of articles of interest that were found across the highest number of Wikipedia language versions. Due to the observed lack of a normal distribution in article access, we used nonparametric methods. We considered a *P* value of <.05 to be statistically significant.

Abnormalities in Wikipedia access patterns that occurred from July 1, 2015, to September 13, 2020, were investigated by using the Chi-square goodness-of-fit test. To identify the qualitative changes in the annual top 10 most accessed Wikipedia Medicine Project articles, we used word clouds as graphical representations of access patterns.

### Identifying Shifts in Search Patterns Before and During the COVID-19 Pandemic

We determined whether the COVID-19 pandemic altered temporal article access patterns. This was achieved by using a k-nearest neighbor (kNN) unsupervised clustering method for analyzing selected Wikipedia language versions. As we were interested in the patterns of access to articles that were of increased user interest in a given period, we performed separate unsupervised clustering analyses for the 2017-2019 and 2020 periods. We excluded the 2015-2016 period due to the possible influence of the Zika virus epidemic, which could have promoted interests in the general population that were similar to interests during the COVID-19 pandemic. As such, we were able to identify articles of user interest during the 2017-2019 and 2020 periods.

We provided a tabular representation of kNN-recognized articles that were associated with increased access before and during the COVID-19 pandemic. The weights that were detected by the kNN algorithm were used to analyze monthly changes in the public’s interest in overrepresented articles during the COVID-19 pandemic and these articles’ relation to the total number of global and regional deaths resulting from SARS-CoV-2 infection. This correlation was tested by performing a Spearman rank correlation analysis. We further divided the data by month (ie, March to September 2020) to evaluate whether the correlation changed between months.

We compared navigation patterns between articles of interest that were published before and during the COVID-19 pandemic by using the DOSs of reference articles. These patterns were tested with the Kruskall-Wallis test, Dunn posthoc test, and *U* Mann-Whitney test.

## Results

### Characteristics of Wikipedia Medicine Project Articles

We analyzed visits to Wikipedia Medicine Project articles that were published in 10 language versions of Wikipedia from 2015 to 2020. These languages included English, French, German, Swedish, Dutch, Russian, Italian, Spanish, Polish, and Vietnamese. We analyzed 100 articles from English Wikipedia after mapping the other language versions of Wikipedia (ie, 100 articles from French Wikipedia, 98 from Dutch Wikipedia, 98 from Spanish Wikipedia, 97 from Russian Wikipedia, 96 from Italian Wikipedia, 95 from German Wikipedia, 94 from Polish Wikipedia, and 93 from Vietnamese Wikipedia). Due to its low coverage (ie, <90 matched articles, as determined via the interwiki mechanism), Swedish Wikipedia was removed from the analysis. Wikipedia articles without coverage across all languages included the following: “Bed bug” (25% coverage), “Coronavirus” (50% coverage), “Adderall” (62.5% coverage), “Bubonic plague” (62.5% coverage), “Chronic traumatic encephalopathy” (62.5% coverage); “Plantar fasciitis” (75% coverage), “Alprazolam” (87.5% coverage), “Cancer” (87.5% coverage), “Elizabeth Holmes” (87.5% coverage), “Escitalopram” (87.5% coverage), “Ketogenic diet” (87.5% coverage), “Lorazepam” (87.5% coverage), “Project MKUltra” (87.5% coverage), “Psychopathy” (87.5% coverage), and “Trypophobia” (87.5% coverage). Full lists of articles that are available for each language are provided in Multimedia Appendix 2, Supplementary Table S1.

In the 2015-2020 period, there were a total of 2,258,621,012 Wikipedia entries in the top 100 most accessed articles and 775,791,941,485 overall visits to Wikipedia across all selected languages (Wikipedia access across all languages: median 0.41%; range 0.29%-0.46%). Annual access to Wikipedia significantly and gradually decreased from 2015 to 2020 (*r*=−0.0513; *P*=.03). Significant increases in use (*P*<.001) were observed for the English (*r*=0.0619), Polish (*r*=0.1237), Dutch (*r*=0.0481), Italian (*r*=0.2197), and Vietnamese (*r*=0.7480) versions of Wikipedia. Significant decreases in use (*P*<.001) were observed for the German (*r*=−0.2654), Spanish (*r*=−0.1085), and Russian (*r*=−0.4338) versions of Wikipedia. For the top 100 most accessed Wikipedia medical articles, we observed a 1.5-fold to 3.8-fold increase in access across all language versions of Wikipedia in 2020 (ie, compared to access in previous years).

### Surge in Article Visits During Early 2020

**Figure 1:**
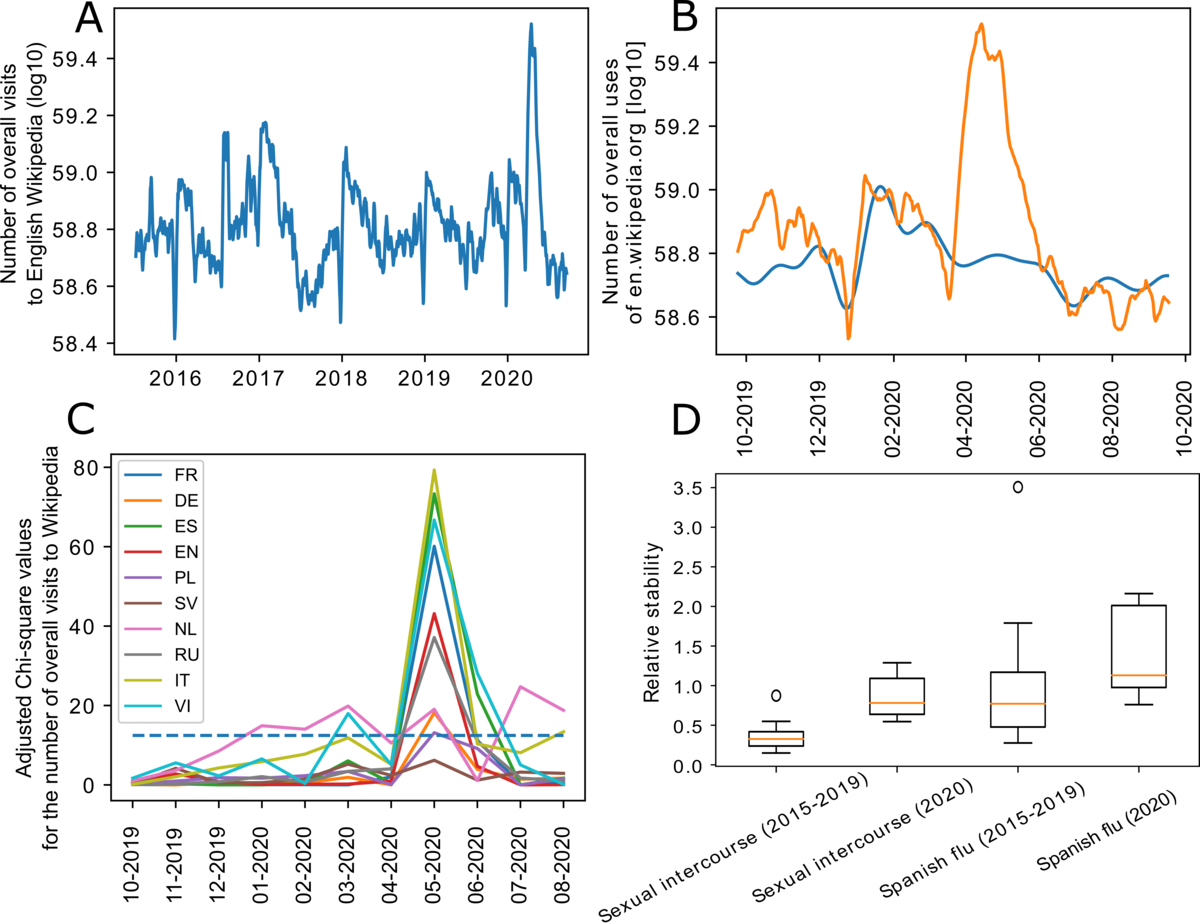
The disruption in annual Wikipedia visit patterns. (A) The pattern of general English Wikipedia access from 2015 to 2020 (Multimedia Appendix 3). Data for other language versions of Wikipedia are provided in Multimedia Appendix 4, Supplementary Figures S1a-S9a. (B) The FBProphet prediction model was created based on data from the 2015-2019 period. The model compared expected behaviors in 2020 (ie, the blue line) to observed access (ie, the orange line). Data for the other language versions of Wikipedia are provided in Multimedia Appendix 4, Supplementary Figures S1b-S9b. (C) A summary of monthly general access to Wikipedia across all Wikipedia language versions from September 2019 to September 2020. Solid lines represent Chi-square goodness-of-fit values and the dashed line represents the cutoff value. (D) The stability of the two reference Wikipedia articles across all languages in the 2015-2019 and 2020 periods (ie, the moving SD divided by mean percent access across a 30-day window). DE: German; EN: English; ES: Spanish; FR: French; IT: Italian; NL: Dutch; PL: Polish; RU: Russian; SV: Swedish; VI: Vietnamese.

To determine the effect of the COVID-19 pandemic on Wikipedia use, we used a graphical representation of total daily Wikipedia access (Figure 1). Due to the suspected increase in Wikipedia use during 2020, we constructed a generalized additive model by using a limited-memory Broyden-Fletcher-Goldfarb-Shanno fitting algorithm that was implemented in the FBProphet tool to predict Wikipedia use in 2020 based on previous access patterns. We based the model on data from the 2015-2019 period to identify expected behaviors in 2020 and compare them to observed access patterns. The global pattern for Wikipedia access was indicative of seasonal behaviors (Figure 1). This pattern changed across all language versions of Wikipedia in March 2020, and it returned to normal in September 2020, as predicted by our models. The Chi-square goodness-of-fit test values for monthly Wikipedia access had *P* values of <.001 across all eight languages (Figure 1).

To determine how abnormal Wikipedia access altered the relative search stability of articles, we needed to pick a point of reference. We determined that the “Sexual intercourse” article demonstrated high yet stable monthly access within the evaluated period. By comparing the “Sexual intercourse” article to the “Spanish flu” article, we were able to determine that the relative access to these articles before the COVID-19 pandemic changed during the pandemic (“Spanish flu” article: *P*<.04; “Sexual intercourse” article: *P*<.001; Figure 1).

### Effects of the COVID-19 Pandemic on the Annual Top 10 Most Commonly Accessed Articles in Wikipedia

We provided a graphical representation of the annual top 10 most accessed articles by creating word clouds for articles that were published from 2015 to 2020 (Multimedia Appendix 3, Multimedia Appendix 4, Supplementary Figures S10-S17). English Wikipedia word clouds are shown in Multimedia Appendix 3, and those for other language versions of Wikipedia are shown in Multimedia Appendix 4, Supplementary Figures S10-S17. We investigated how the 10 most viewed articles changed each year. In the 2015-2019 period, we observed a set of articles that were constantly among the top 10 most accessed articles each year. These articles were as follows: “Leonardo da Vinci” (appearance frequency: 37/45), “Asperger syndrome” (appearance frequency: 36/45), and “Bipolar disorder” (appearance frequency: 28/45). Compared to previous years, there was a distinctive difference in the top 10 most accessed articles in 2020; the “Pandemic” (appearance frequency: 9/9), “COVID-19 pandemic” (appearance frequency: 9/9), “Coronavirus disease 2019” (appearance frequency: 8/9), and “Spanish flu” (appearance frequency: 8/9) articles became the most frequently accessed articles. We found 8 new articles among the top 10 most accessed articles in 2020 that have never appeared in this list. Of these articles, 2 were created after the SARS-CoV-2 pandemic (ie, the “COVID-19 pandemic” and “Coronavirus disease 2019” articles), while the other 6 were present within Wikipedia before the pandemic (ie, the ”Pandemic,” “Spanish flu,” “Coronavirus,” “Bubonic plague,” ”Influenza,” and “World Health Organization” articles).

### Identifying Cross-Language Similarities in Article Access Prior to and During the COVID-19 Pandemic via Unsupervised Clustering

The across-language abnormality that was observed in Wikipedia and Wikipedia Medicine Project access frequency in 2020 was further investigated. By conducting an unsupervised analysis, we identified differences between article access before and during the COVID-19 pandemic. Articles that were published in 2020 and recognized by the kNN algorithm were considered COVID-19–related articles. These included the following eight articles: “Coronavirus,” “Spanish flu,” “Coronavirus disease 2019,” “COVID-19 pandemic, “Influenza,” “Pandemic,” “Virus,” and “Black Death.” The group of articles that were unrelated to COVID-19 included 25 articles. We selected the four articles from this group that were present in most language versions of Wikipedia (ie, the “Sexual intercourse,” “Leonardo da Vinci,” “Bipolar disorder,” and “Borderline personality disorder” articles).

We compared the DOSs of COVID-19–related articles to the DOSs of articles that were unrelated to COVID-19. We used the “COVID-19 pandemic” article as a point of reference for COVID-19–related articles and the “Sexual intercourse” article as a point of reference for articles unrelated to COVID-19. The analysis showed that the DOSs between articles unrelated to COVID-19 and the “Sexual intercourse” article were significantly higher than the DOSs between COVID-19–related articles and the “COVID-19 pandemic” article (Kruskall-Wallis test and posthoc Dunn test: *P*<.001; Table 1). This indicated that COVID-19–related articles were more closely connected, which we suspected due to the observed similarities in themes.

We performed a Spearman rank correlation analysis on articles to provide additional context. We observed that articles unrelated to COVID-19 had higher median correlation values across languages than those in the COVID-19–related article group (Table 1). In the COVID-19–related article group, the highest median correlation value (ie, across all languages) was found between the “COVID-19 pandemic” and “Coronavirus disease 2019” articles (R=0.7022), and the lowest median correlation value was found between the “COVID-19 pandemic” and “Influenza” articles (R=0.0330). In the group of articles unrelated to COVID-19, correlation values were higher. The highest median correlation value was found between the “Sexual intercourse” and “Bipolar disorder” articles (R=0.8657), and the lowest median correlation value was found between the “Sexual intercourse” and “Leonardo da Vinci” articles (R=0.4631).

We also compared the DOSs between all articles that were analyzed in detail and the “COVID-19 pandemic” article. COVID-19–related articles had significantly lower DOSs compared to articles unrelated to COVID-19 (*U* Mann-Whitney test: *P*<.001; Table 1).

**Figure 2:**
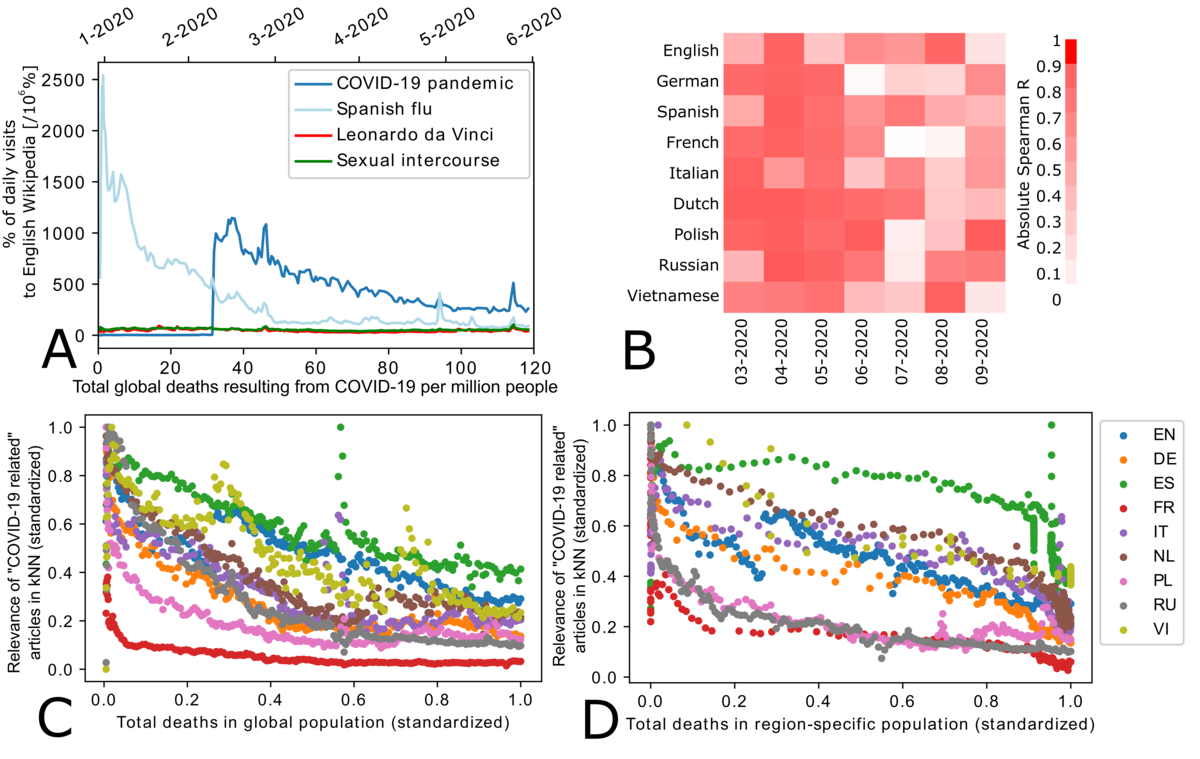
Access to COVID-19–related Wikipedia articles in 2020 and the total number of deaths resulting from SARS-CoV-2 infection. (A) The percentage of daily article access (ie, the "COVID-19 pandemic," "Spanish flu," "Leonardo da Vinci," and "Sexual intercourse" articles) to English Wikipedia and total number of global deaths resulting from SARS-CoV-2 infection (ie, per 1 million people). Data for other language versions of Wikipedia are provided in Multimedia Appendix 4, Supplementary Figures S18-S25. (B) Heatmap of Spearman absolute regression coefficients for COVID-19–related articles and the total number of global deaths resulting from SARS-CoV-2 infection across languages and months. (C) Access to COVID-19–related articles (ie, the kNN-determined relevance values across selected Wikipedia language versions) versus the total number of region-specific deaths resulting from SARS-CoV-2 infection. The graph shows correlations between region-specific deaths and COVID-19–related articles. (D) Access to COVID-19–related articles (ie, the kNN-determined relevance values across selected Wikipedia language versions) versus the total number of global deaths resulting from SARS-CoV-2 infection. DE: German; EN: English; ES: Spanish; FR: French; IT: Italian; kNN: k-nearest neighbor; NL: Dutch; PL: Polish; RU: Russian; SV: Swedish; VI: Vietnamese.

Finally, we investigated how the patterns in articles access (ie, those identified by unsupervised clustering) were associated with the total number of global and region-specific deaths resulting from SARS-CoV-2 infection. To this end, we standardized each measure. We used ordinary least squares linear regression to identify correlations across each month in the March to September 2020 period (Figure 2, Multimedia Appendix 2, Supplementary Table S3).

Upon further investigation, we found that the kNN-derived pattern in COVID-19–related articles’ access was significantly associated with both the total number of global and region-specific deaths resulting from SARS-CoV-2 infection for articles across all Wikipedia language versions (Figure 2). There was a notable difference in the correlation between article access and the total number of deaths resulting from SARS-CoV-2 infection, which appeared linear for region-specific deaths and negatively exponential for global deaths (Figure 2). This was also reflected by the low absolute Spearman rank coefficients between article access and the total number of global deaths resulting from SARS-CoV-2 infection for the months after June 2020 (Figure 2).

**Table 1:** Degrees of separation (DOSs) between investigated articles and reference articles within and across article clusters for the 2016-2019 and 2020 periods.

Wikipedia articles			Prevalence^a^, %		Spearman R^b^ (within groups)		DOSs within groups^c^ (IQR)		DOSs across articles^d^ (IQR)
**COVID-19–related article group**									
	Coronavirus	80		0.2770		1 (1-1)		1 (1-1)	
	COVID-19 pandemic	77.8		Reference^e^		Reference^e^		Reference^e^	
	Spanish flu	55.6		0.4451		2 (1.75-2)		2 (1.75-2)	
	Coronavirus disease 2019	55.6		0.7022		1 (1-1.25)		1 (1-1.25)	
	Pandemic	44.4		0.4341		1 (1-1)		1 (1-1)	
	Black death	33.3		0.4332		2 (2-2)		2 (2-2)	
	Virus	11.1		0.3851		2 (1-2)		2 (1-2)	
	Influenza	11.1		0.0330		1.5 (1-2)		1.5 (1-2)	
**Group of articles unrelated to COVID-19**									
	Sexual intercourse	88.9		Reference^e^		Reference^e^		3 (2-3)	
	Leonardo da Vinci	88.9		0.4631		2 (1.5-2)		2 (2-2.5)	
	Bipolar disorder	77.8		0.8657		3 (2-3)		3 (2.75-3)	
	Borderline personality disorder	77.8		0.8352		2.5 (2-3)		3 (3-3)	

^a^Refers to the presence of an article in k-nearest neighbor–determined groups across all available languages.

^b^The Spearman R value for an article.

^c^The DOS of an article within a k-nearest neighbor–determined group.

^d^The DOS across all articles that were investigated in detail.

^e^The article of reference for calculating DOSs.

## Discussion

### Principal Results

Our study shows that the pandemic has significantly influenced patterns in Wikipedia access. There was an apparent surge in interest for infectious disease, which somewhat surprisingly and quickly declined despite the mounting death toll. This is the first study on the impact of the previously recognized change in society’s interest, which resulted from the COVID-19 pandemic. In this study, this impact was reflected by changes in Wikipedia access patterns. Moreover, the analysis of different Wikipedia language versions allowed us to confirm that the observed effect of these changes is reflected by both the regional and global impacts of the COVID-19 pandemic. Our study provides proof that an unsupervised clustering method for analyzing data on daily access to medical information could be used to identify interests in global health issues.

Recent studies have indicated that the global lockdown is a possible reason for the change in internet use [28]. We noticed a pronounced increase in the total number of visits to Wikipedia in March 2020. Not only did we observe more frequent visits to Wikipedia, but we also observed a distinctive change in searched topics. The list of the top 10 most accessed articles in 2020 included articles that have not appeared in such lists during previous years. Moreover, the DOS between most of the articles of interest was low, suggesting that navigation was relevant to the observed change. This finding, combined with the correlations between article access, indicated that during the pandemic, people had an increased interest in topics that were not directly related to COVID-19 but were related to pandemics in general. Correlations in access to the “Spanish flu,” “Black Death,” and “Bubonic plague” articles show that the general public has rapidly gained interest in the topic of previous infectious disease–related health crises. We suspect that these observed correlations are associated with the COVID-19 Wikiproject, which curates articles to provide users with valuable information. The COVID-19 Wikiproject, which is managed by 1200 editors, has resulted in the addition of more than 6500 entries to Wikipedia [29].

Our study shows that similar changes in public interests have already occurred in recent years. We noticed an increase in access to the 2016 “Zika virus” article in 5 of the 9 investigated language versions of Wikipedia. This could be associated with the epidemic that was announced by the WHO in November 2016. This association was also confirmed in the Bragazzi et al [30] and Hickmann et al [31] studies on H1N1-related and Zika virus–related articles and these viruses’ respective outbreaks. Other studies have focused on the increased amount of searches for terms related to anosmia (ie, a characteristic symptom of COVID-19) and have shown its correlation with the SARS-CoV-2 outbreak in many countries [32]. Moreover, trends in the amount of searches for the terms “wash hands,” “hand sanitizer,” and “antiseptic” have successfully predicted the rising number of COVID-19 cases in many countries, which indicates that search trends can potentially be used in epidemiologic surveillance [33]. In our analysis, we noticed an increase in the popularity of a Wikipedia article about the WHO. This result may indicate that the general public has decided to learn more about the organization, making the WHO a focal point for the outbranching of searches. Conversely, this increase in popularity could be linked with President Donald Trump’s attempts to discredit the WHO for the way that they handled the COVID-19 outbreak. These attempts were highly amplified by mainstream media and Twitter [34].

We were interested in identifying the aspect of the pandemic that specifically drove people’s growing interest in COVID-19 from March to June 2020. Our unsupervised clustering analysis showed that the total number of global deaths significantly correlated with the temporal increase in the number of searches for COVID-19–related articles (ie, from March to June 2020). Interestingly, despite the continuous rise in the number of global deaths, the number of visits to COVID-19–related articles decreased after June 2020. This could be interpreted as a gradual decline in global interest toward the pandemic. Data on interest in COVID-19–related articles suggest that there is an increased interest in contagious diseases and that an opportunity to raise awareness through Wikipedia-centered strategies will arise during future outbreaks of infectious diseases. However, the stable correlation between the region-specific number of deaths and the frequency of searches for COVID-19–related articles that persisted until September 2020 is a noteworthy finding. A study by Gozii et al [35] showed that public attention and response are mostly driven by media coverage instead of disease spread. Interestingly, they also showed that the media sharply shifted their focus toward domestic situations as soon as the first death was confirmed in a person’s home country [35]. Therefore, we conclude that reporting about region-specific death reshapes individuals’ perceptions of risk and significantly impacts public interest, thereby affecting the information-seeking behaviors of people from affected areas.

Our study's limitations include the fact that our analysis was restricted to article access data, which did not contain detailed information on user-specific article access patterns or users’ demographic characteristics (eg, age, gender, and educational status). However, we refrained from performing such analyses to maintain the privacy of Wikipedia users. Our study proves that it is possible to identify the global and regional effects of a health crisis on people's behavior. The in-depth analysis of users’ behaviors can be unethical due to its potential for targeting region-specific populations and spreading disinformation, which are ever-present concerns due to the malicious spread of fake news.

Language bias may have also slightly skewed our results, as preferences for English may have resulted in an underreporting of native language–specific searches. Moreover, several idiomatic phrases that are specific to the English vocabulary—“black death” being the prime example—may not directly translate to other languages. To mitigate this bias, we assumed that analyzing data from English Wikipedia would account for most of the global population’s language preferences. Further, we used search data from the other language versions of Wikipedia to validate our results. This strategy yielded a cohesive and surprisingly uniform sample of COVID-19–related search data.

### Conclusions

Our results support the idea that Wikipedia can be used as a tool for successfully surveilling trends in public interest. The increase in interest toward COVID-19–related articles was followed by a progressive decline. This shows that the potential optimal window for efficient information dissemination via Wikipedia is the early phase of a pandemic. Wikipedia articles that directly link to major articles about a major health crisis can contribute to the spread of global anxiety and the promotion of prevention behaviors. Therefore, Wikipedia articles should be carefully selected.

## Appendix

Multimedia Appendix 1 Open data.

Multimedia Appendix 2 Supplementary tables S1-S3.

Multimedia Appendix 3 Word cloud.

Multimedia Appendix 4 Supplementary figures S1-S25.

## Abbreviations

DOS: degree of separation
kNN: k-nearest neighbor
PHP: hypertext preprocessor
WHO: World Health Organization
