# Correction: Assessing Public Interest Based on Wikipedia’s Most Visited Medical Articles During the SARS-CoV-2 Outbreak: Search Trends Analysis

**DOI:** 10.2196/29598

**Published:** 2021-04-15

**Authors:** Jędrzej Chrzanowski, Julia Sołek, Wojciech Fendler, Dariusz Jemielniak

**Affiliations:** 1 Department of Biostatistics and Translational Medicine Medical University of Łódź Łódź Poland; 2 Department of Pathology Medical University of Łódź Łódź Poland; 3 Management in Networked and Digital Societies Kozminski University Warszawa Poland

In “Assessing Public Interest Based on Wikipedia’s Most Visited Medical Articles During the SARS-CoV-2 Outbreak: Search Trends Analysis” (J Med Internet Res 2021;23(4):e26331) the authors noted two errors.

Due to a system error, the name of one author, *Wojciech Fendler*, was replaced with the name of another author on the paper, *Dariusz Jemielniak*. In the originally published paper, the order of authors was listed as follows:

Jędrzej Chrzanowski; Julia Sołek; Dariusz Jemielniak; Dariusz Jemielniak

This has been corrected to:

Jędrzej Chrzanowski; Julia Sołek; Wojciech Fendler; Dariusz Jemielniak

In the originally published paper, the ORCID of author 'Wojciech Fendler' was incorrectly published as follows:

Wojciech Fendler: 0000-0002-3745-7931

This has been corrected to:

Wojciech Fendler: 0000-0002-5083-9168

The correction will appear in the online version of the paper on the JMIR Publications website on April 15, 2021, together with the publication of this correction notice. Because this was made after submission to PubMed, PubMed Central, and other full-text repositories, the corrected article has also been resubmitted to those repositories.

