# Supplementary material for: Assessing Public Interest Based on Wikipedia’s Most Visited Medical Articles During the SARS-CoV-2 Outbreak: Search Trends Analysis
Source: J Med Internet Res. 2021 Apr 12;23(4):e26331. doi: 10.2196/26331 (PMC8049630; doi:10.2196/26331)
Supplement: Multimedia Appendix 3 [file jmir_v23i4e26331_app3.pdf]

1

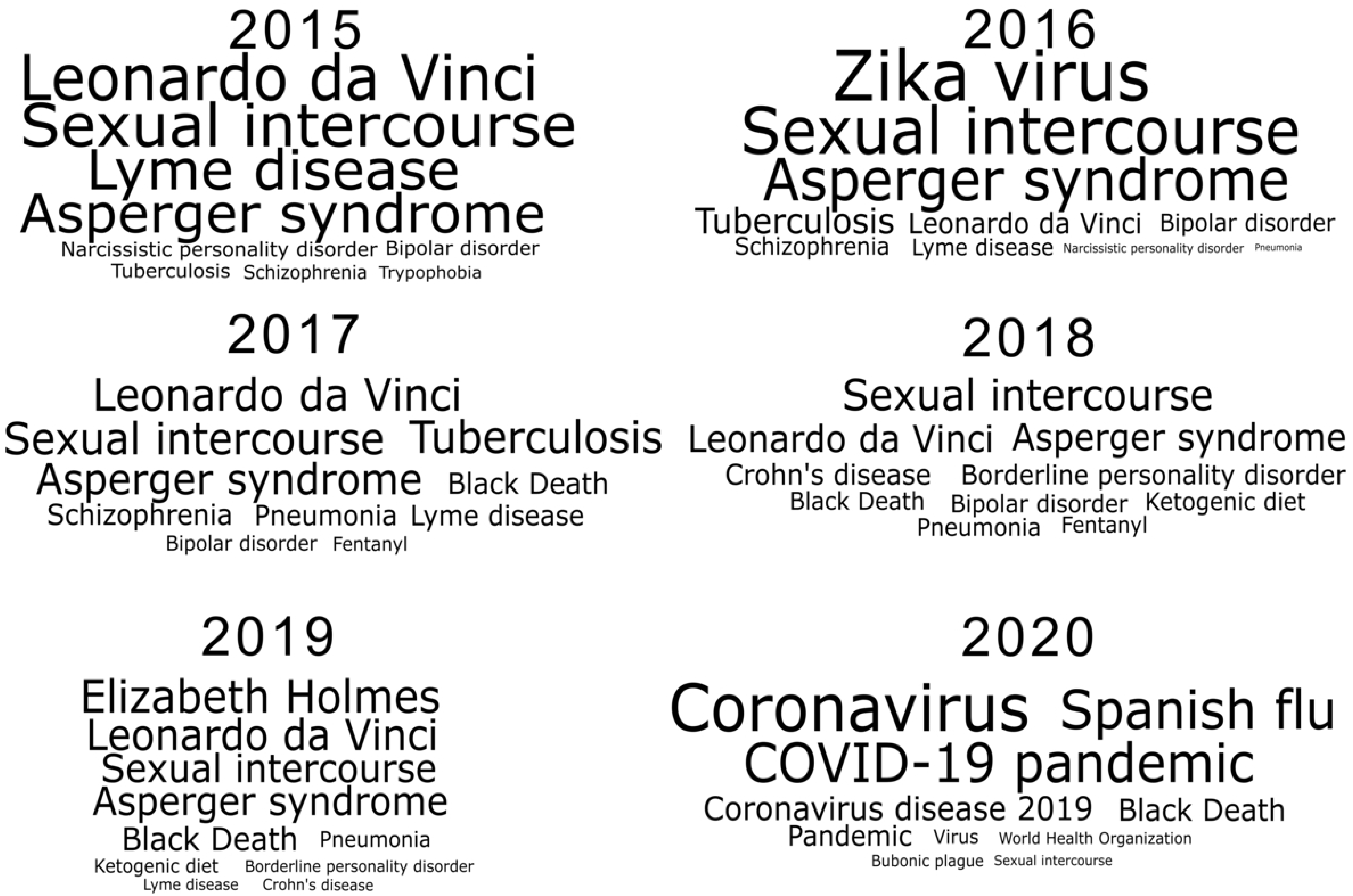
2015
Leonardo da Vinci
Sexual intercourse
Lyme disease
Asperger syndrome
Narcissistic personality disorder
Bipolar disorder
Tuberculosis
Schizophrenia
Trypophobia
2016
Zika virus
Sexual intercourse
Asperger syndrome
Tuberculosis
Leonardo da Vinci
Bipolar disorder
Schizophrenia
Lyme disease
Narcissistic personality disorder
Pneumonia
2017
Leonardo da Vinci
Sexual intercourse
Tuberculosis
Asperger syndrome
Black Death
Schizophrenia
Pneumonia
Lyme disease
Bipolar disorder
Fentanyl
2018
Sexual intercourse
Leonardo da Vinci
Asperger syndrome
Crohn's disease
Borderline personality disorder
Black Death
Bipolar disorder
Ketogenic diet
Pneumonia
Fentanyl
2019
Elizabeth Holmes
Leonardo da Vinci
Sexual intercourse
Asperger syndrome
Black Death
Pneumonia
Ketogenic diet
Borderline personality disorder
Lyme disease
Crohn's disease
2020
Coronavirus
Spanish flu
COVID-19 pandemic
Coronavirus disease 2019
Black Death
Pandemic
Virus
World Health Organization
Bubonic plague
Sexual intercourse
