# Supplementary material for: Assessing Public Interest Based on Wikipedia’s Most Visited Medical Articles During the SARS-CoV-2 Outbreak: Search Trends Analysis
Source: J Med Internet Res. 2021 Apr 12;23(4):e26331. doi: 10.2196/26331 (PMC8049630; doi:10.2196/26331)

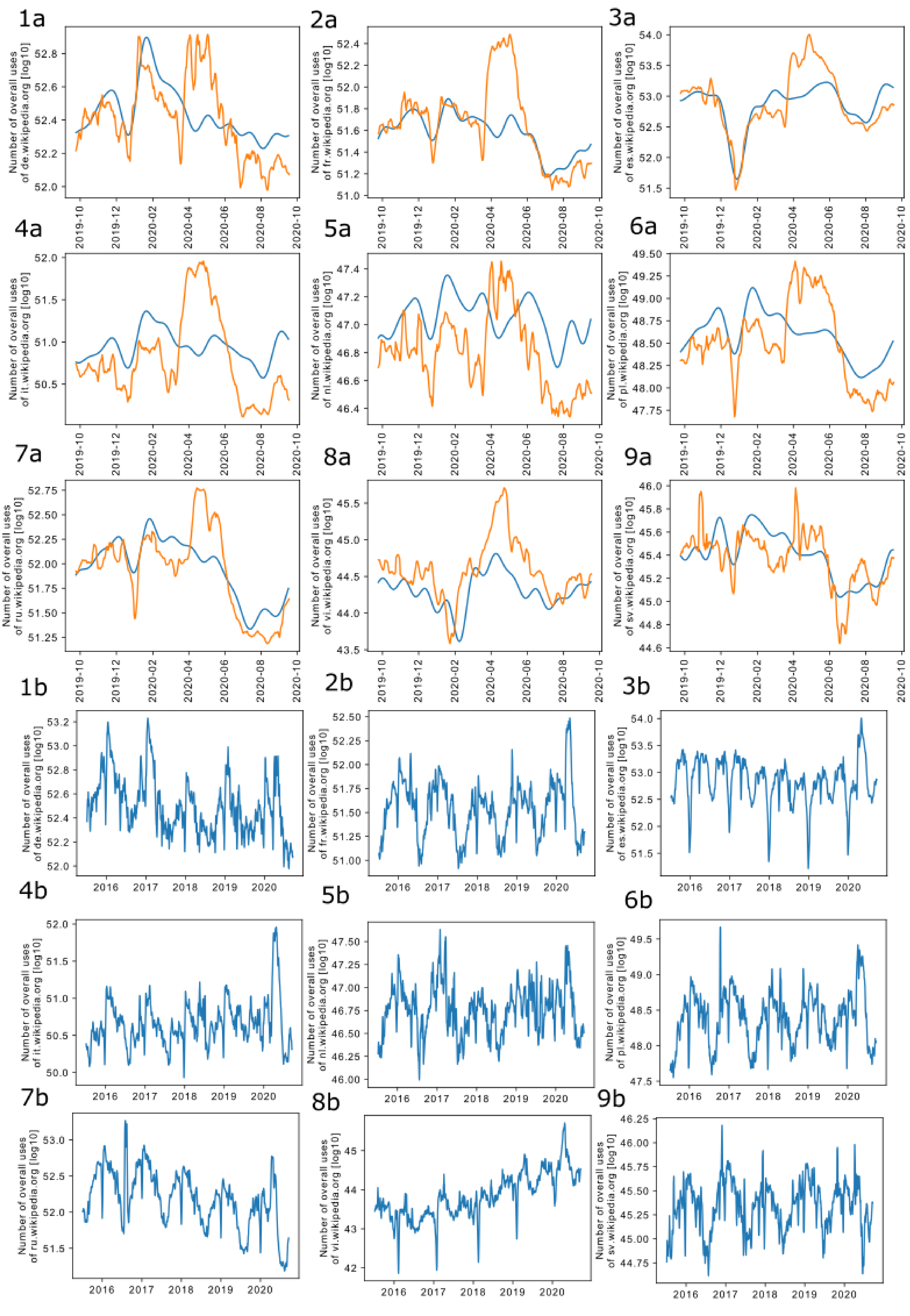
1a
Number of overall uses of de.wikipedia.org [log10]
2a
Number of overall uses of fr.wikipedia.org [log10]
3a
Number of overall uses of es.wikipedia.org [log10]
4a
Number of overall uses of it.wikipedia.org [log10]
5a
Number of overall uses of nl.wikipedia.org [log10]
6a
Number of overall uses of pl.wikipedia.org [log10]
7a
Number of overall uses of ru.wikipedia.org [log10]
8a
Number of overall uses of vi.wikipedia.org [log10]
9a
Number of overall uses of sv.wikipedia.org [log10]
2019-10
2019-12
2020-02
2020-04
2020-06
2020-08
2020-10
1b
Number of overall uses of de.wikipedia.org [log10]
2b
Number of overall uses of fr.wikipedia.org [log10]
3b
Number of overall uses of es.wikipedia.org [log10]
4b
Number of overall uses of it.wikipedia.org [log10]
5b
Number of overall uses of nl.wikipedia.org [log10]
6b
Number of overall uses of pl.wikipedia.org [log10]
7b
Number of overall uses of ru.wikipedia.org [log10]
8b
Number of overall uses of vi.wikipedia.org [log10]
9b
Number of overall uses of sv.wikipedia.org [log10]
2016
2017
2018
2019
2020

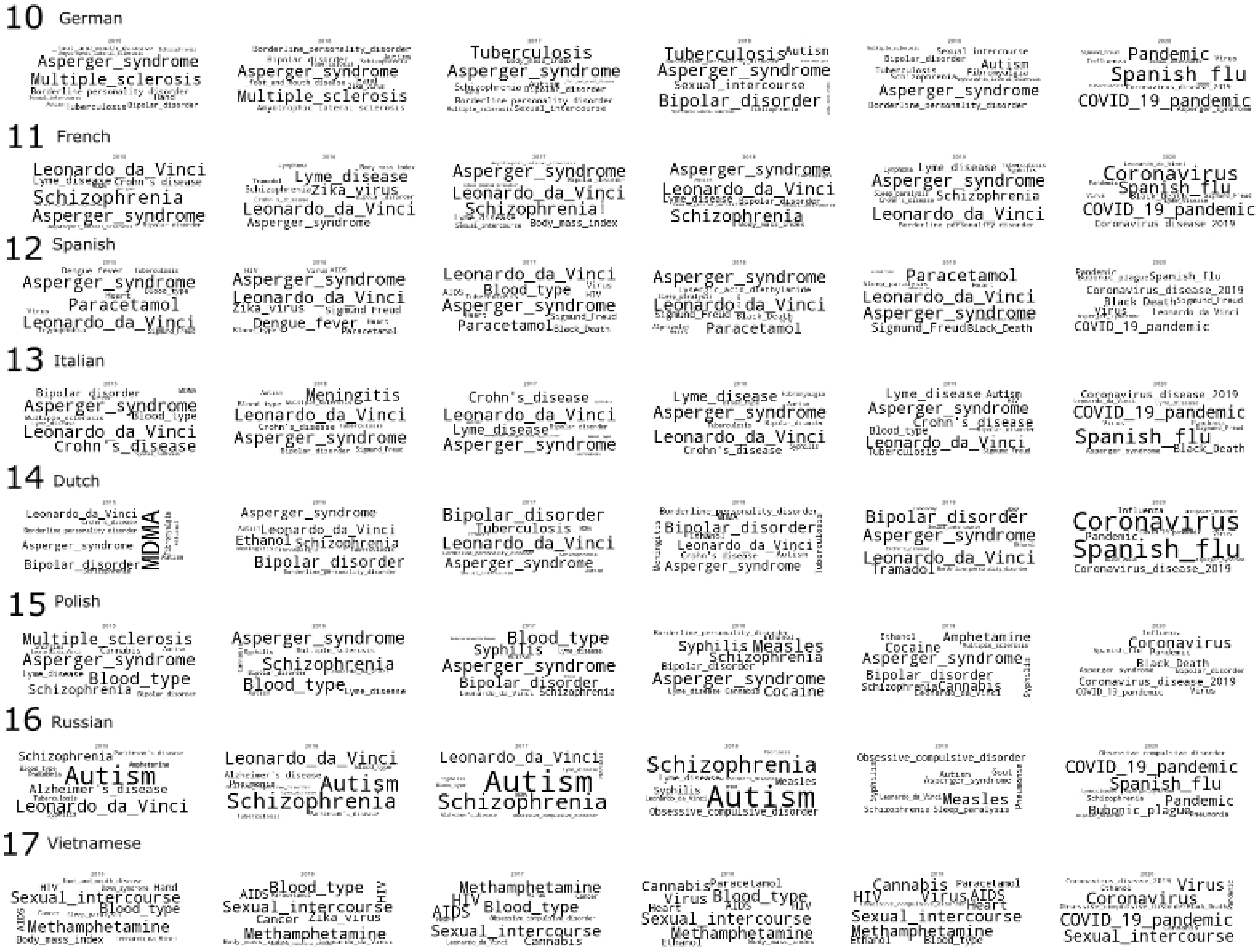
10 German
11 French
12 Spanish
13 Italian
14 Dutch
15 Polish
16 Russian
17 Vietnamese

18

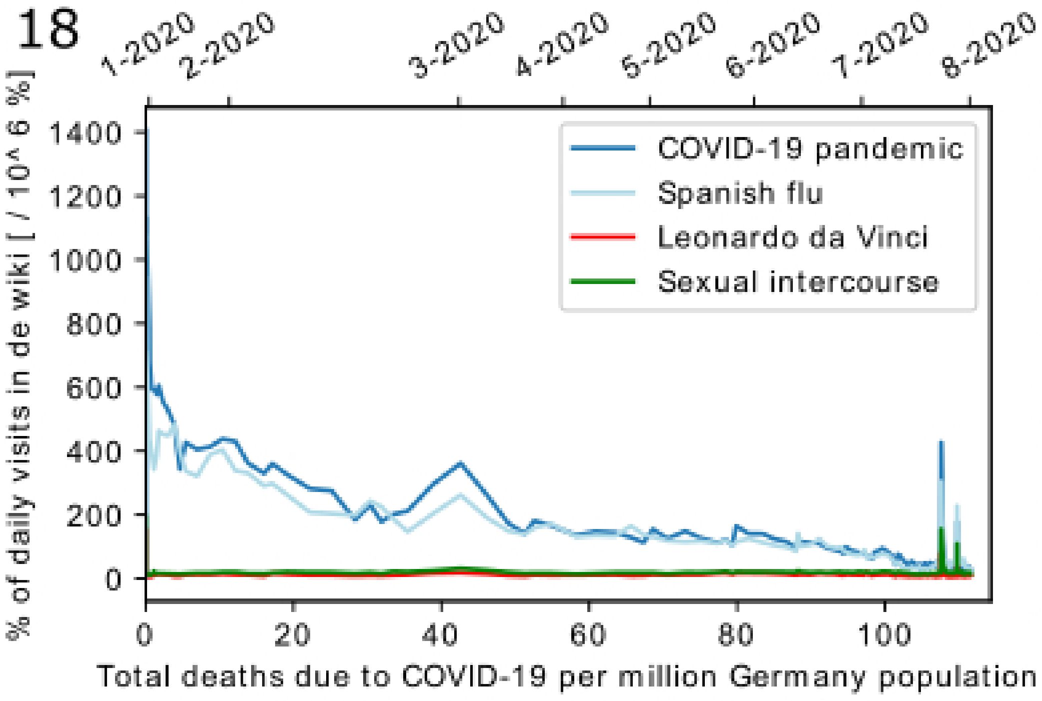

19

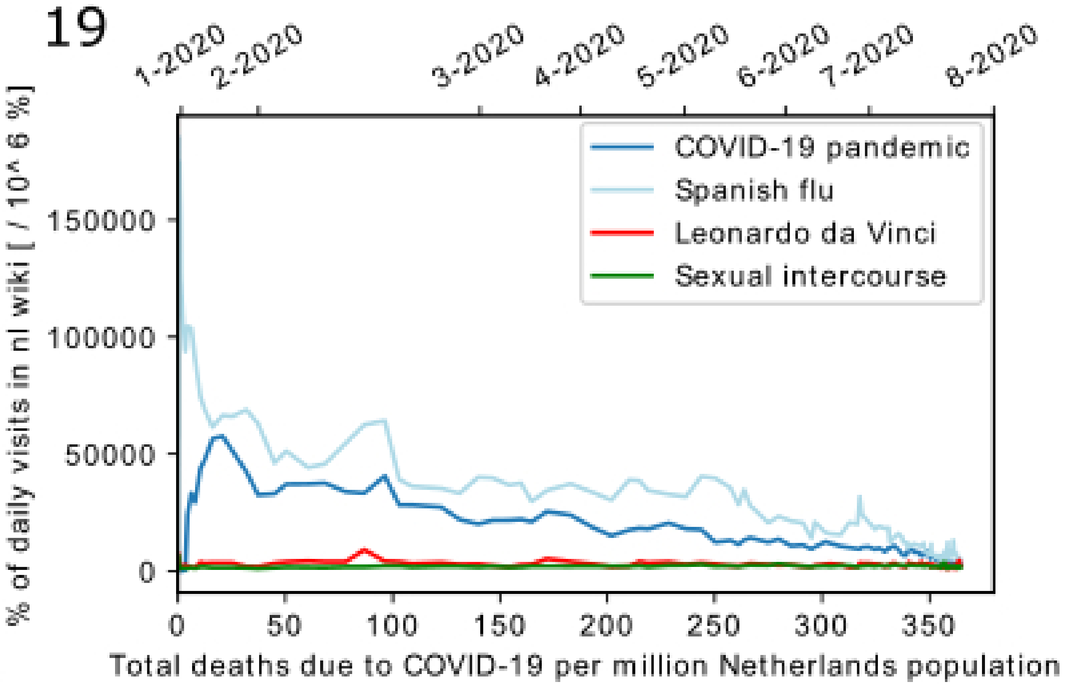

20

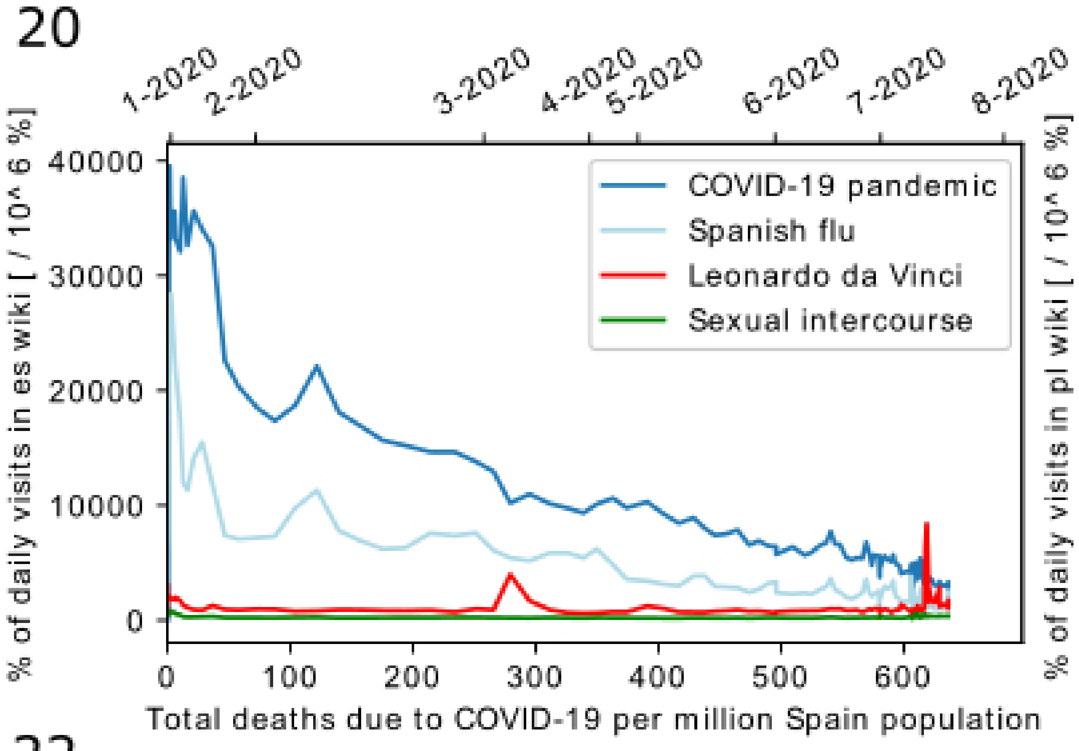

21

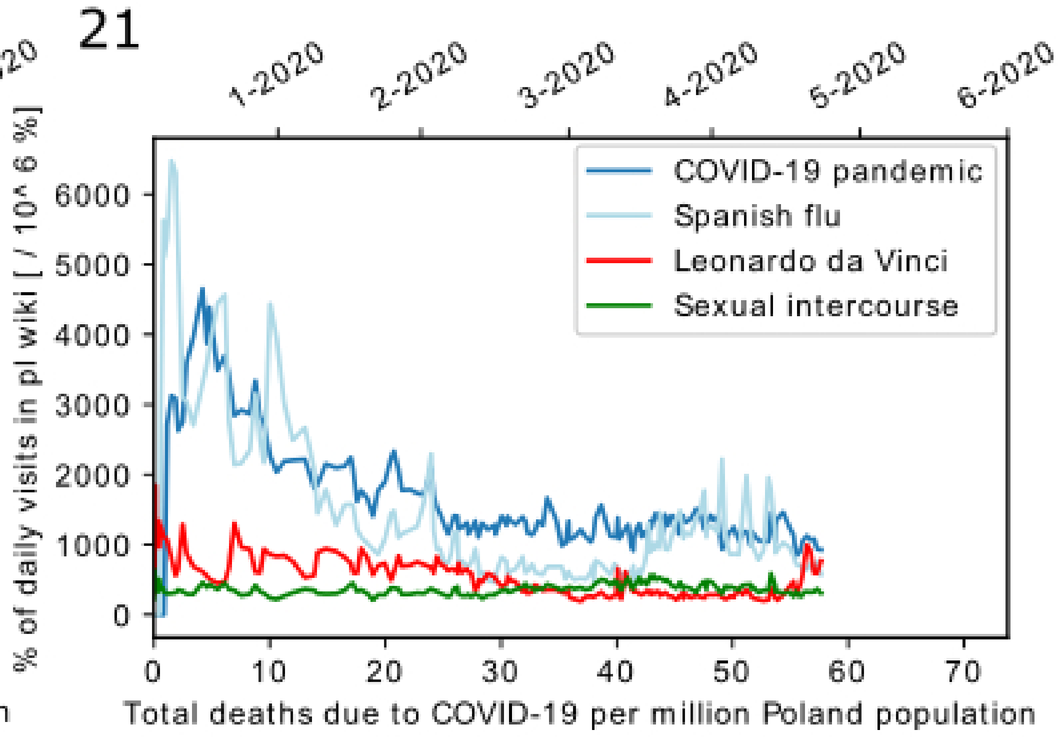

22

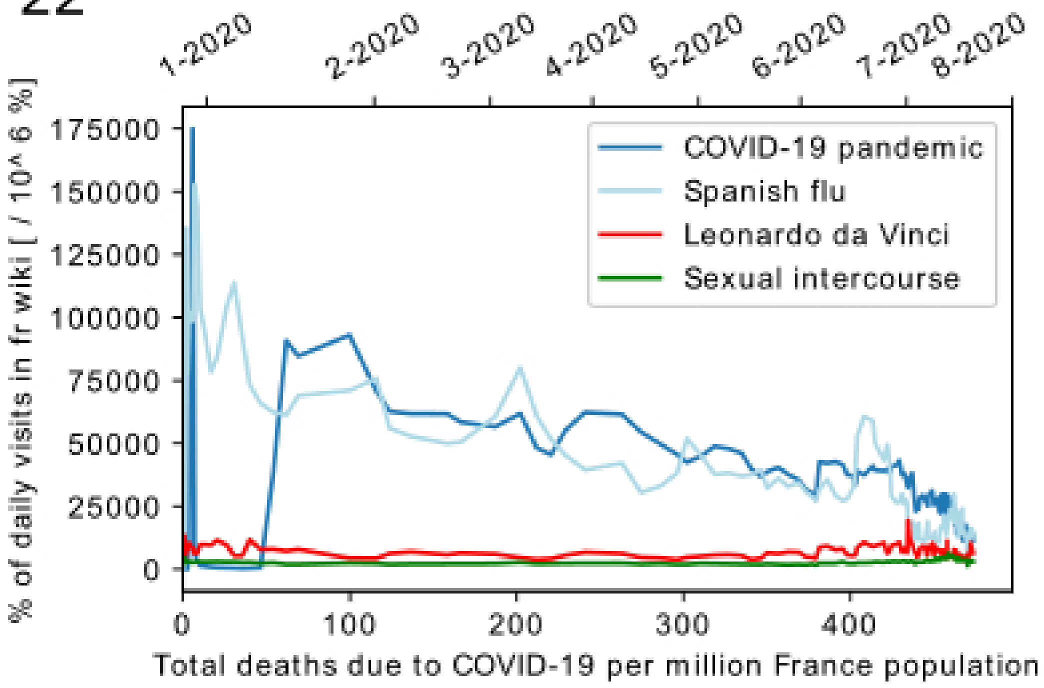

23

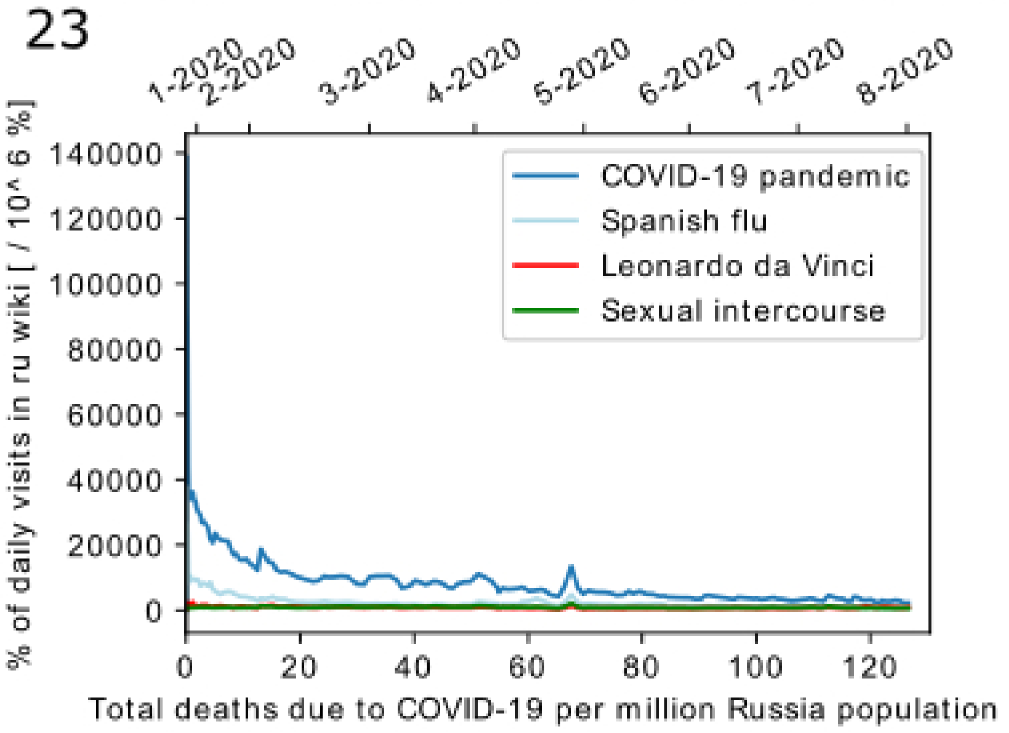

24

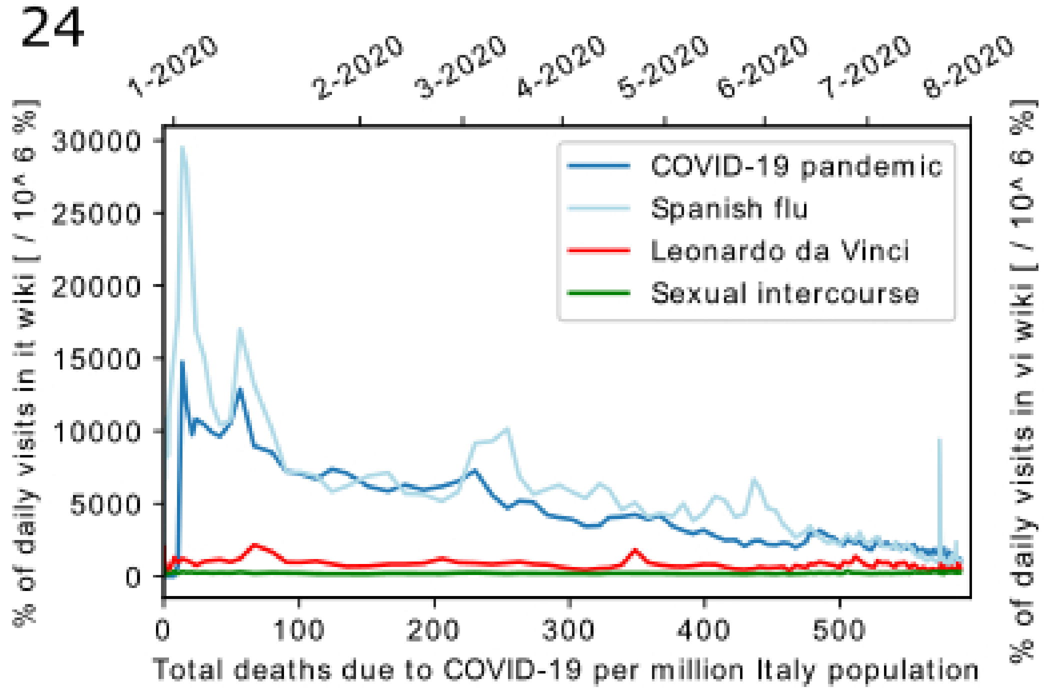

25

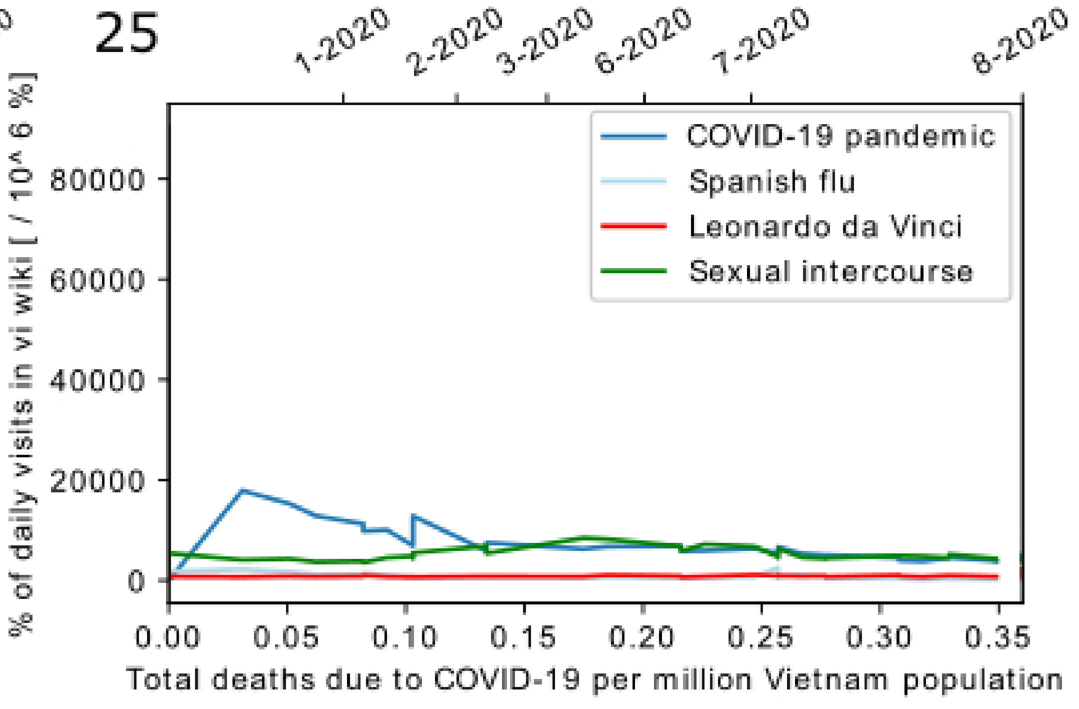

Supplement: Multimedia Appendix 4 [file jmir_v23i4e26331_app4.pdf]
